# Development of a Three-Dimensional Bioengineered Platform for Articular Cartilage Regeneration

**DOI:** 10.3390/biom10010052

**Published:** 2019-12-28

**Authors:** Gerard Rubí-Sans, Lourdes Recha-Sancho, Soledad Pérez-Amodio, Miguel Ángel Mateos-Timoneda, Carlos Eduardo Semino, Elisabeth Engel

**Affiliations:** 1 Biomaterials for Regenerative Therapies group, Institute for Bioengineering of Catalonia (IBEC), The Barcelona Institute of Science and Technology, 08028 Barcelona, Spain; grubi@ibecbarcelona.eu (G.R.-S.); sperez@ibecbarcelona.eu (S.P.-A.); mamateos@ibecbarcelona.eu (M.Á.M.-T.); 2 CIBER en Bioingeniería, Biomateriales y Nanomedicina (CIBER-BBN), Barcelona, Spain; 3 Tissue Engineering Laboratory, IQS School of Engineering, Ramon Llull University, 08017 Barcelona, Spain; lourdes.recha@gmail.com; 4 Department of Materials Science and Metallurgical Engineering, EEBE campus, Technical University of Catalonia (UPC), 08019 Barcelona, Spain; 5 Hebe Biolab S.L., C/Can Castellvi 27, 08017 Barcelona, Spain

**Keywords:** polycaprolactone, 3D printing, RAD16-I self-assembling peptide, chondrogenic differentiation

## Abstract

Degenerative cartilage pathologies are nowadays a major problem for the world population. Factors such as age, genetics or obesity can predispose people to suffer from articular cartilage degeneration, which involves severe pain, loss of mobility and consequently, a loss of quality of life. Current strategies in medicine are focused on the partial or total replacement of affected joints, physiotherapy and analgesics that do not address the underlying pathology. In an attempt to find an alternative therapy to restore or repair articular cartilage functions, the use of bioengineered tissues is proposed. In this study we present a three-dimensional (3D) bioengineered platform combining a 3D printed polycaprolactone (PCL) macrostructure with RAD16-I, a soft nanofibrous self-assembling peptide, as a suitable microenvironment for human mesenchymal stem cells’ (hMSC) proliferation and differentiation into chondrocytes. This 3D bioengineered platform allows for long-term hMSC culture resulting in chondrogenic differentiation and has mechanical properties resembling native articular cartilage. These promising results suggest that this approach could be potentially used in articular cartilage repair and regeneration.

## 1. Introduction

Cartilage pathologies such as osteoarthritis, injuries or aging affect a large percentage of the world’s population. Poor regenerative properties of this tissue, due to the absence of an intrinsic mesenchymal stem cell (MSC) population [1,2], and an inefficient response by the human body when healing cartilage injuries, leads to calcification and ossification, causing severe pain and loss of functionality. Articular cartilage is an avascular tissue covering bone ends in articulating joints formed by chondrocytes and a complex extracellular matrix (ECM), where collagen type II, aggrecan proteoglycan and multiadhesive proteins are the main components [3,4]. This tissue, provides low friction elastic surfaces able to withstand dynamic compressive loads several times higher than body weight [5,6]. This behavior can be attributed to the complex biochemical properties and physical structure of the cartilage ECM produced by chondrocytes [7].

Traditional articular cartilage repair strategies such as arthroscopic microfracture or autologous chondrocyte implantation cannot effectively heal the affected area and only joint replacement provides a permanent solution [8,9,10]. Cartilage tissue engineering is aimed at creating artificial constructs substituting and regenerating this damaged tissue with long-lasting biomanufactured replacement tissues [4,11,12]. Most strategies incorporate three main components: (1) a biocompatible and biodegradable scaffold; (2) either chondrocytes or MSCs able to differentiate, and (3) a combination of bioactive signaling molecules to mimic the native tissue environment in order to induce tissue regeneration [9,13]. These scaffolds are typically three-dimensional structures manufactured from synthetic and/or natural polymers that provide mechanical, physical and biochemical support for cells. Although these structures can mimic the mechanical properties of cartilage, drawbacks exist in recreating this complex structure as well as in promoting and maintaining cell metabolic processes that externally produce joint tissue [14]. In this study, we combined the three different components to provide a 3D platform that can not only mimic the mechanical properties of cartilage, but also produce the native tissue in vitro.

To produce a biocompatible and biodegradable scaffold, we introduced RAD16-I, a short, water soluble (Ac-RADARADARADARADA-NH2) self-assembling peptide (PuramatrixTM, BD, Erembodegem, Belgium) [15,16]. RAD16-I self-assembly is promoted by electrostatic interactions on the hydrophilic side of the peptide fiber and hydrophobic interactions on the other side, in addition to the β-sheet hydrogen bonds along the backbone [17]. This 3D hydrogel provides a nanofiber environment permeable to small molecules such as gases, nutrients and growth factors. Its mechanical strength can be controlled by adjusting peptide concentration, and RAD16-I has proven biocompatibility and biodegradability [15]. RAD16-I nanofibres can also be used to encapsulate cells providing a truly 3D environment [15] without any toxic effects [18]. RAD16-I was also shown to maintain a chondrocyte phenotype and promote biosynthesis of cartilage ECM when used as a scaffold for tissue-engineered cartilage [19]. Furthermore, in previous studies, a PCL/RAD16-I composite scaffold induced redifferentiating potential to dedifferentiated human articular chondrocytes [20].

To provide a tailored scaffold for patient-specific cartilage and osteochondral tissue formation, 3D printing is a promising technique, as it produces structures matching in shape and size the existing defects [21,22,23,24]. Among the different materials used in 3D printing, polycaprolactone (PCL) is a key material due to its biocompatibility and printability, slow degradation rate, high permeability and stiffness [25,26,27], and excellent rheological and viscoelastic properties [28]. Moreover, it is an excellent raw material for scaffold fabrication, as it mimics mechanical properties of articular cartilage [29,30]. Moreover, PCL does not influence cell phenotypes and allows cell proliferation, adhesion and migration processes to take place [31].

By combining 3D printed PCL constructs with RAD16-I self-assembling peptide we developed a novel three-dimensional bioengineered platform providing a macrostructure capable of withstanding the same mechanical forces as the human cartilage and, a nanostructure which enables MSCs to proliferate, migrate and carry out metabolic processes such as differentiation into chondrocytes and articular cartilage ECM production. The obtained PCL/RAD16-I scaffold populated by human MSCs has a promising future application in tissue engineering of cartilage through high expression of key chondrogenic biomarkers and mimicking the mechanical properties of native cartilage.

## 2. Materials and Methods

### 2.1. Materials

Polycaprolactone (6-caprolactone polymer, #440744, PCL) with average Mn of 80,000 kDa was purchased from Sigma–Aldrich (Saint Louis, MO, USA) and RAD16-I self-assembling peptide, commercially known as Corning^®^ PuramatrixTM Peptide Hydrogel (#45354250) was purchased from Corning Inc. (New York, NY, USA). Human bone marrow mesenchymal stem cells were obtained from patients and kindly donated by Texas A&M Health Science Center College of Medicine Institute for Regenerative Medicine at Scott & White (NIH Grant P40RR017447).

### 2.2. Methods

#### 2.2.1. Scaffold Design and 3D-Printing Process

A PCL solution (30% *w*/*w* in chloroform, CHCl3) was used for 3D scaffold printing in 3-mL syringes using 0.20 mm inner diameter tips (TIP 27 GA 0.2 × 6.35 mm, #7018395, Nordson EFD©). A direct nozzle-deposition system (Direct print tool, Tissue Engineering 3Dn-300 Sciperio/nScrypt, Orlando, FL, US) was employed for 3D printing. The printing parameters were set at 40 PSI printing pressure, constant speed of 3 mm/s the surface-tip distance (≈100 µm) was manually adjusted for appropriate material deposition. Chloroform was evaporated overtime and totally removed by sinking scaffolds three times in 20 mL 70% ethanol and milliQ water baths under agitation. As a result, 70 × 12 × 2 mm rectangular grids of printed PCL were obtained with a theoretical pore size of 300 µm. Finally, 8 mm diameter, round shaped scaffolds were obtained cutting PCL grids with punches (Harris Uni-coreTM, Altadena, CA, USA).

#### 2.2.2. Micro-Computed Tomography (µCT)

3D reconstructions were obtained using a µCT equipment (Skyscan 1076, Bruker, Kontich, Belgium). Acquisition conditions were the following: image resolution 9 µm, 40 kV of voltage, 250 µA of current. Images were taken without any filter; n = 3.

#### 2.2.3. Scaffold Hydrolyzation

Scaffolds were hydrolyzed overnight in 10 mL of 4 M sodium hydroxide (NaOH) solution under agitation.

#### 2.2.4. Contact Angle Measurements

Water contact angle (WCA) was measured with a goniometer (DSA 100; Kruss, Hamburg, Germany) at room temperature (RT) using Milli-Q water and RAD16-I 0.5% (*w*/*v*). Drops were deposited over non-treated and treated 3D printed PCL scaffolds to determine the hydrophobic properties of the constructs after hydrolyzation process. Three replicates were performed (n = 3).

#### 2.2.5. 2D and 3D Culture of Human Mesenchymal Stem Cells (hMSCs)

##### 2D Culture

Human mesenchymal stem cells (hMSCs) from bone marrow (BM) were seeded at a density of 10,000 cells/cm^2^ and expanded in T175 flasks with expansion medium consisting of: Alpha Minimum Essential medium (α-MEM; Invitrogen/GIBCO, Carlsbad, CA, USA), Fetal Bovine Serum (FBS, 16% *v*/*v*; Atlanta Biologicals, Flowery Branch, GA, USA), L-Glutamine 100× (1% *v*/*v*; Labclinics, Barcelona, Spain), Penicillin/Streptomycin 100× (1% *v*/*v*; Labclinics, Barcelona, Spain). Cultures were kept at 37 °C in a 5% CO_2_ humidified environment.

##### 3D Cell Seeding and Culture

hMSCs at passage 6 were seeded on 3D printed PCL scaffolds, RAD16-I self-assembling peptide encapsulations and on scaffolds fabricated by combining both PCL/RAD16-I.

For PCL scaffolds, 5 × 10^5^ cells were suspended in 80 µL of cell culture medium and added to scaffolds, as 80 µL correspond to the internal volume of scaffolds. After 1 h, culture medium was added to reach a final volume of 800 µL/well.

For RAD16-I self-assembling peptide scaffolds, 1.6 × 10^5^ cells were suspended in sucrose 10% *w*/*v* with a 0.3% *v*/*v* RAD16-I solution for a final 80 µL volume, resulting in a final concentration of 0.15% *v*/*v* of the self-assembling peptide, as this is known to be the most effective for cell culture [32,33,34]. To seed the cell-self-assembling peptide suspension, 150 µL of culture medium were added to 48-well plates. After 1 h, culture medium was added to reach a final volume of 800 µL/well. Differences in cell seeding density with other conditions are due to the impossibility of encapsulating higher cell number in RAD16-I self-assembling peptide while keeping the peptide plus cells volume constant at 80 µL. 

For PCL/RAD composite scaffolds, 5 × 10^5^ cells were suspended in sucrose 10% *w*/*v* and RAD16-I at a final concentration of 0.25% *v*/*v* in a total volume of 80 µL and seeded in PCL scaffolds in 48-well plates. This peptide concentration was determined to provide maximum stiffness values in order to obtain cartilage tissue-like characteristics for cell processes [34,35]. After cell seeding, 200 µL of culture medium were added in order to induce RAD16-I self-assembly. After 1 h, culture medium was added to reach a final volume of 800 µL/well.

#### 2.2.6. Cell Proliferation and Cytotoxicity Assays

MTT [3-(4,5-dimethylthiazolyl-2)-2,5-diphenyltetrazolium bromide] (M5655; Sigma, Saint Louis, MO, USA) assay reagent was used to examine cell metabolic activity related to cell proliferation and cytotoxicity (ISO 10993-5:2009) by measuring the absorbance of formazan as the insoluble product of MTT reduction by cells. Culture medium was removed from wells and MTT reagent was added at a concentration of 0.5 mg/mL in culture medium. 3D samples were incubated for 3 h at 37 °C in darkness. Then, scaffolds and RAD16-I encapsulations were placed in 800 µL of dimethyl sulfoxide (DMSO, D8418; Sigma, Saint Louis, MO, USA) for 3 h at RT in dark conditions. Conditioned expansion medium from PCL, PCL/RAD and RAD scaffolds was used for cytotoxicity assays following ISO 10993-5:2009 standard. Scaffolds were kept 24 h in expansion media. Next, 10^4^ hMSC were seeded in 96-well plates and cultured for 24, 48 and 72 h with conditioned medium from each scaffold condition. Non-conditioned media was used as control. Samples were analyzed per triplicate. Absorbance was read at 550 nm in a microplate reader (ELX808; Biotek, Winooski, VT, USA).

#### 2.2.7. Scanning Electron Microscopy (SEM)

Construct morphology and physical properties were observed under scanning electron microscope (NOVA NanoSEM 230, FEI Company, Hillsboro, OR, USA). Samples were fixed one hour in PFA 1% and dried sequentially in ethanol baths (20, 40, 60, 80, 96 and 100%). Then samples underwent a supercritical drying process followed by a carbon coating and were analyzed under SEM microscope at 5 kV. Three replicates were analyzed for each condition (n = 3).

#### 2.2.8. Dynamic Mechanical Analysis (DMA)

A compression assay with DMA Multi-Frequency-Strain mode and a frequency sweep test was performed with a DMA Q800 (TA Instruments, Inc, New Castle, DE, USA) to PCL and PCL/RAD cultured scaffolds, RAD encapsulations and human, chicken and calf native cartilage. Results were compared to 3D PCL scaffolds non-treated with NaOH and not cultured.

Assay parameters were 2 µm of amplitude (0.1 of the total scaffold thickness), 0.01 N of preload force and a constant frequency of 1 Hz, whereas construct diameter and thickness were ≈8 mm and ≈2 mm.

Results of storage modulus (G’, elastic behavior), loss modulus (G’’, viscous response), complex modulus (G*, addition of storage and loss modulus) and tan delta (ratio between loss and storage modulus) were obtained and analyzed using TA Instruments, Inc. software. Three replicates were analyzed per condition (n = 3).

#### 2.2.9. Chondrogenic Differentiation

Differentiation of hMSCs towards the chondrogenic lineage was assessed in cells cultured in expansion and in chondrogenic culture medium. Chondrogenic medium consists of Dulbecco’s Modified Eagle’s Medium GlutaMAX (DMEM GlutaMAX, Gibco, Carlsbad, CA, USA), Penicillin/Streptomycin 100× (1% *v/v*; Labclinics, Barcelona, Spain), 40 μg/mL L-Proline (Sigma), 1 mM Sodium Pyruvate (Life Technologies, Carlsbad, CA, USA), ITS Premix 100× (1× *v/v*; Insulin, human transferrin and selenous acid; BD Bioscience, Allschwil, Switzerland), 10 ng/mL recombinant human Transforming Growth Factor-β1 (TGF-β1; Millipore), 25 μg/mL L-Ascorbic Acid 2-phosphate (AA2P; Sigma) and 100 nM Dexamethasone (Sigma). Cell culture medium was changed every 2 days by renewing half of the volume for 30 days.

##### Real-time Reverse Transcriptase-Polymerase Chain Reaction (qRT-PCR)

Chondrogenic and hypertrophic gene expression was quantified by real-time reverse transcriptase polymerase chain reaction. Three-dimensional constructs were washed with PBS and stored at −20 °C in RTL buffer. Qiagen RNeasy^®^ Plus Mini Kit (74134, Qiagen, Barcelona, Spain) was used to purify RNA from samples. RNA purity and concentration were quantified using a NanoDrop Spectrophotometer (ND-1000, NanoDrop^®,^ De Meern, the Netherlands) and then converted into cDNA using a RT2 First Strand Kit (330404, Qiagen). The obtained cDNA was analyzed by real-time reverse transcriptase polymerase chain reaction (qRT-PCR) using RT2 SYBR Green ROX qPCR Mastermix (330524, Qiagen) and primers were designed for each of the genes. The primers used were the following: Human Beta Actin (ACTB, PPH00073G-200, Qiagen) as the housekeeping gene, collagen type I (COL1A1), collagen type II (COL2A1), collagen type X (COL10A1), SOX9, a transcription factor required for the expression of COL2A1, aggrecan (ACAN), and RUNX2, a hypertrophic transcription factor (all human, primer sequences are shown in Table 1). Human RNA (Human XpressRef Universal Total RNA, QIAGEN) was used as a positive and interplate control to compensate for variations between plates. Real-time PCR was carried out under the following conditions: 10 min at 95 °C followed by 40 cycles of 15 s at 95 °C and 1 min at 60 °C. Finally, a melting curve was performed on the plates, first for 15 s at 95 °C, followed by 1 min at 60 °C and the melting step from 60 to 95 °C. Relative gene-fold variations were determined by the 2^-ΔΔCt^ method using beta actin as the housekeeping gene. Three replicates were analyzed per condition (n = 3).

##### Western Blot

3D constructs were lysed using RIPA buffer (R0278; Sigma) mixed with protease inhibitor cocktail (Complete Mini; 11836153001; Roche, Barcelona, Spain) and crushed by twisting and grinding with micropestles. Total protein concentration was quantified by Pierce BCA Protein assay kit (23225; Thermo Fisher Scientific, Waltham, MA, USA). Next, 5 mg of protein from 3D constructs were run in 7.5% acrylamide gels applying a voltage of 150 V for 1 h approximately.

After running proteins in an electrophoresis tank, these were transferred to nitrocellulose membranes (0.45 µm) (162-0115; BIO-RAD, Hercules, CA, USA) by applying 100 V for 1 h at 4 °C. Afterwards, membranes were incubated at RT for 2 h in blocking buffer (BB) consisting of 5% (*w*/*v*) non-fat powdered milk in phosphate buffered saline with 0.1% tween (PBST).

Membranes were incubated overnight at 4 °C with primary antibodies at a final concentration of 1 mg/mL in PBST. Then, secondary antibodies horseradish peroxidase (HRP) conjugated were added, in a concentration of 1 mg/mL, and incubated at RT for 2 h. Finally, membranes were revealed for HRP detection with ClarityTM Western ECL Substrate (170-5061; BIO-RAD). Chemiluminescent images were taken in the ImageQuantTM LAS 4000 mini (GE HealthCare).

Primary antibodies used were anti-collagen I (ab6308; Abcam, Cambridge, UK), anti-collagen II (MA5-12789; Thermo Fisher Scientific), anti-collagen X (ab182563; Abcam), anti-actin (sc-1615; Santa Cruz Biotechnology). Secondary antibodies used were anti-mouse IgG-HRP (ab97023; Abcam), anti-rabbit IgG-HRP (ab97051; Abcam) and anti-goat IgG-HRP (ab97100; Abcam). Three replicates were analyzed per condition (n = 3).

##### Immunohistochemistry

Histological evaluation of cartilage ECM formation and chondrogenic markers expression was performed staining samples using immunohistochemical techniques. Cell cultured 3D constructs were OCT (4583, Tissue-Tek^®^, Barcelona, Spain) embedded and samples were gradually frozen to –80 °C. Histological cuts were done at 20 µm thickness in a cryostat (Leica CM1900).

Immunochemical and hematoxylin staining methods were performed to observe hypertrophic and chondrogenic markers (collagen I and II) and nuclei stained. Non-specific interactions were blocked using 4% *v*/*v* Goat serum. As the secondary antibodies were HRP conjugated, endogenous peroxidase was quenched with 3% H2O2 solution in PBS to avoid its presence after 3,3’-Diaminobenzidine (DAB, Sigma–Aldrich) incubation. Samples were incubated with primary (overnight at 4 °C) and secondary (1h at RT) antibodies and then 3 min DAB incubation was performed. Finally, nuclei were stained with hematoxylin 2%, tissue sections were dehydrated with 95% ethanol, 100% ethanol and xylene wells (×2) and Eukitt mounting medium (Panreac Eukitt^®^) was used to preserve samples with coverslips (D102460, Deltalab, Rubí, Spain).

Primary antibodies used were anti-collagen I (ab6308; Abcam) and anti-collagen II (MA5-12789; Thermo Fisher Scientific). Secondary antibody used was anti-mouse IgG-HRP (ab97023; Abcam). Negative controls were performed without primary antibodies. Three replicates were analyzed per condition (n = 3).

#### 2.2.10. Statistics

Obtained results were expressed as mean *±* SD. Statistical differences were assessed with GraphPad Prism 6 (La Jolla, CA, USA) carrying out one- or two-way ANOVA tests, followed by Tukey post analysis. Statistical significance was considered at a value of α < 0.05, and represented as follows: *p < 0.05, **p < 0.01, ***p < 0.001, ****p < 0.0001; n = 3.

## 3. Results

### 3.1. 3D-Printed Scaffold Characterization

3D-printed PCL scaffolds were obtained by pneumatically extruding prewarmed 30% PCL in chloroform through 200 µm diameter nozzles. Printing pressure and speed (40 PSI, 3 mm/s) were studied to effectively print large scaffolds, from which 8 mm diameter and 2 mm height disks were cut. To ensure complete chloroform removal from scaffolds, these were exposed to air for solvent evaporation followed by displacement with several 70% ethanol and water baths.

Scaffolds’ wettability was measured by water contact angle resulting in an average of 111.70° *±* 6.48° for the untreated scaffolds, due to PCL being a highly hydrophobic polymer. To reduce scaffolds’ overall hydrophobicity, they were hydrolyzed overnight in 4 M NaOH to expose carboxylic groups on PCL surface. After hydrolyzation, no contact angle measurements could be taken, as water and RAD16-I peptide drops were able to penetrate the structure.

**Figure 1:**
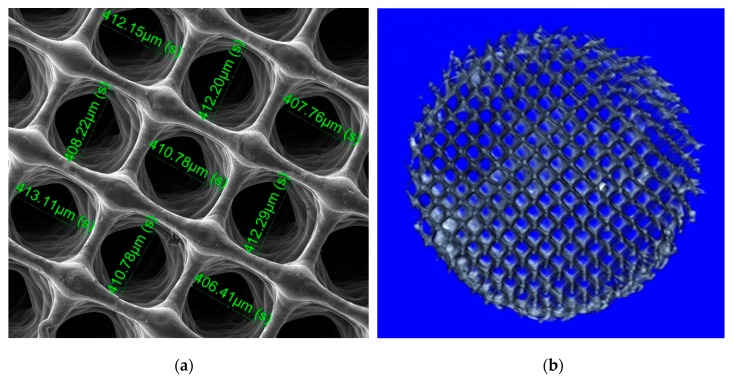
Determination of scaffold porosity. (**a**) SEM image taken to measure fiber diameter and pore size after the printing process. N = 3. Scale bar = 500 µm. (**b**) Non-cultured PCL scaffold three-dimensional reconstruction and sectioning to observe the different physical properties. n = 3. Scale bar = 1 mm.

Following the scaffolds printing process and hydrolyzation, their structure and porosity were assessed using scanning electron microscopy (SEM) and microcomputed tomography (µCT) (Figure 1).

The printing design included 200 µm printed PCL fiber diameter, which following chloroform removal decreased by 41.3% to an average fiber diameter of 82.7 *±* 12.3 µm. On the other hand, the originally designed pore size of 300 µm was increased a 37.5% to a final pore size of 412.4 *±* 25.7 µm (Figure 1a,b).

Scaffold porosity images obtained with µCT allowed to analyze scaffolds’ internal structure. Vertical and lateral scaffold porosity were properly built during printing and preserved after chloroform removal processes as shown in Figure 1b.

### 3.2. Assessment of Material Cytotoxicity and Cell Metabolic Activity within the Constructs

**Figure 2:**
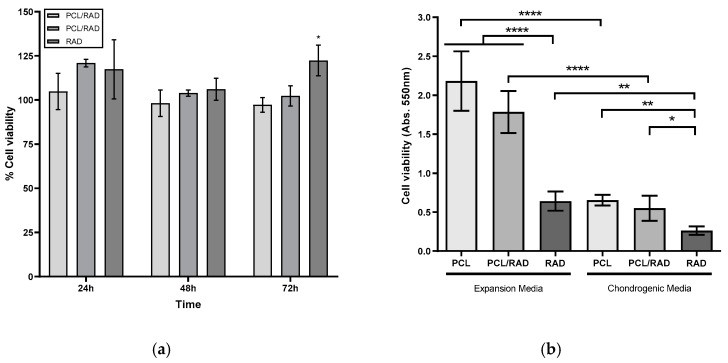
MTT assay for material cytotoxicity and metabolic activity. (**a**) PCL, PCL/RAD and RAD cytotoxicity determination after 24, 48 and 72 h. MTT values were expressed in number of cells. (**b**) Cellular metabolic activity results from hMSCs’ 30-day culture. MTT values were expressed as a measure of formazan (product) absorbance at 550 nm. (Statistical differences are indicated as: *p < 0.05, **p < 0.01, ***p < 0.001 and ****p < 0.0001, N = 3, one-way ANOVA). n = 3.

In order to assess complete chloroform removal from scaffolds, an indirect cytotoxicity assay following the ISO 10993-5:2009 guideline was performed. Hydrolyzed PCL scaffolds, PCL/RAD composites and RAD peptide encapsulations were incubated with cellular expansion medium for a 24 h period. This medium was then added to hMSCs and cultured for 24, 48 and 72 h. Controls consisted of cells cultured in fresh medium. At each timepoint, MTT assay was performed. Results showed no significant differences in metabolic activity between cells treated with conditioned and control medium for all timepoints. After 72 h, significant increase in cell proliferation was observed in cells treated with RAD encapsulations’ conditioned medium (Figure 2a).

Following 30 days of hMSCs culture in PCL, PCL/RAD or RAD scaffolds, in either expansion or chondrogenic media, cell viability and metabolic activity related to cell proliferation were assessed using MTT assay. The hMSCs cultured in expansion medium had significantly higher levels of metabolic activity than when cultured in chondrogenic medium (Figure 2b). Both PCL scaffolds and the composite PCL/RAD scaffolds showed significantly increased cell metabolic activity when compared to RAD16-I only encapsulated cells (which could be due to the initial cell seeding number, 5 × 10^5^ cell per PCL and PCL/RAD scaffold, 1.6 × 10^5^ cells per RAD encapsulation). There were no significant differences between the two scaffolds. Higher metabolic activity was observed in expansion medium due to high serum content (16% FBS) compared to the differentiation medium, in which stimulatory factors such as TGF-β or insulin [36,37] were used to promote chondrogenic differentiation rather than cell proliferation.

### 3.3. Cell-Cultured Scaffolds: Morphological Properties and Extracellular Matrix Deposition

**Figure 3:**
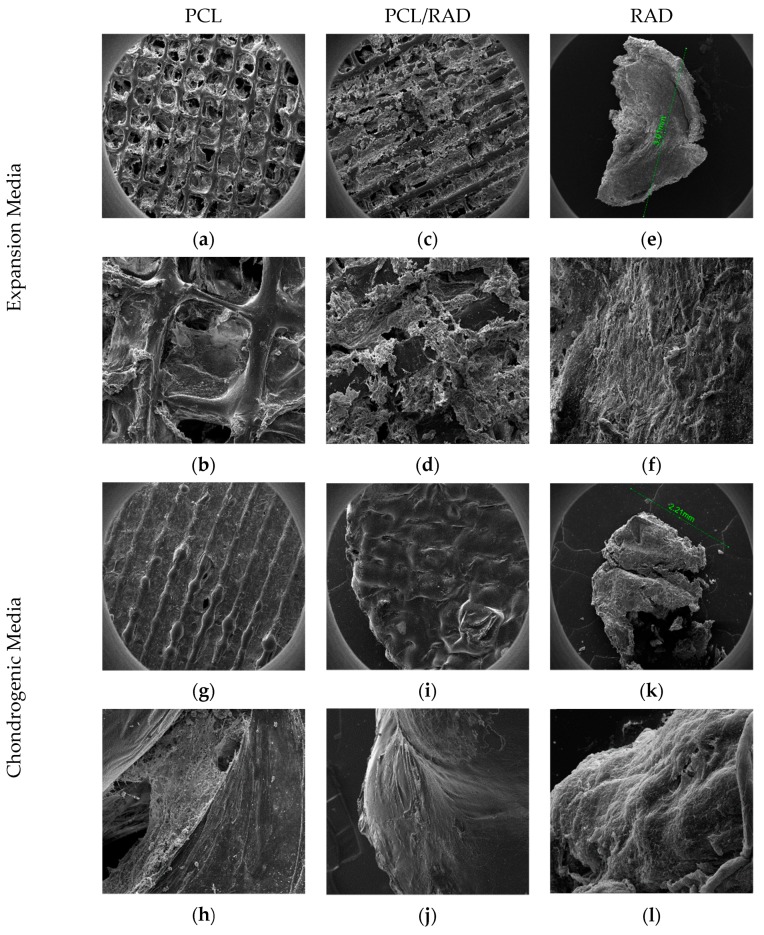
Composite of SEM images from PCL, PCL/RAD and RAD. (**a**–**f**) PCL, PCL/RAD and RAD samples cultured in expansion medium. (**g**–**l**) PCL, PCL/RAD and RAD samples cultured in chondrogenic medium. Two pictures per sample are displayed. Scale bar images a, c, e, g, i and k = 1 mm, images b and d = 300 µm, image j = 100 µm and images f, h and l = 50 µm. n = 3.

Scanning electron microscopy was used to assess the scaffolds’ topography, morphology and internal structure. Following 30-day hMSCs culture, PCL scaffolds in expansion medium showed that hMSCs did not penetrate throughout the entire scaffold’s internal structure (Figure 3a,b). hMSCs used PCL fibers as anchoring points to form cell aggregates but no ECM deposits were observed. hMSC encapsulated in RAD16-I in expansion medium contracted the scaffold from an initial size around 6 mm to a final size of about 3 mm (Figure 3e,f). The self-assembling peptide encapsulations had aligned cells at the surface of the construct, but no ECM deposits were observed (Figure 3f). PCL/RAD composites in expansion medium showed a higher amount of matter filling throughout the scaffold, indicating the positive effect of incorporation of RAD16-I peptide to PCL (Figure 3c,d). RAD16-I peptide covers the PCL scaffold fibers together with embedded cells. However, no ECM deposition was observed, but only the presence of RAD16-I and cells (Figure 3c,d).

ECM deposition was observed in all samples cultured in the chondrogenic medium (Figure 3g–k). In PCL scaffolds, cells were observed to use scaffold fibers as anchor points, not as a surface (Figure 3h). In RAD16-I cell encapsulations, the final construct size was reduced to around 2.2 mm compared to the 6 mm initial size (Figure 3k,l). As previously described, chondrogenic medium promotes cell differentiation, which led to a higher degree of self-assembling polymer contraction by its degradation from cells, cell–cell extensive network formation and cell contraction processes producing also a global contraction [20,32,38]. Unlike RAD16-I samples cultured with expansion medium, higher magnification images of chondrogenic RAD16-I samples showed the presence of cells surrounded by a non-oriented, non-fiber-shaped substance, which corresponds to deposited ECM (Figure 3k,l).

Finally, the PCL/RAD scaffolds cultured in chondrogenic medium (Figure 3i,j) show denser scaffold surfaces and internal structure due to high levels of ECM deposited by cells during the 30-day culture period, and the presence of RAD16-I self-assembling peptide fibers.

### 3.4. Mechanical Properties of Constructs

Viscoelastic mechanical properties of 30-day cell-cultured PCL/RAD scaffolds were assessed and compared to PCL and RAD alone, to native cartilage samples and to non-sodium hydroxide treated nor cell-cultured PCL scaffolds.

The elasticity of samples is defined by the storage modulus, the viscosity by loss modulus and the resistance to deformation by the complex modulus. There were no significant differences between all samples except for RAD samples in both media conditions (Figure S1). This indicates that both scaffold treatment and RAD16-I peptide deposition inside PCL structure did not modify the overall construct mechanical properties. There were no significant differences between PCL and PCL/RAD samples when compared to native cartilage even there is a trend suggesting that this second group has higher elastic properties. These results demonstrate that PCL polymer has appropriate mechanical properties for tissue regeneration approaches, as its viscoelastic behavior is similar to the one from cartilage tissue. RAD samples both in expansion and chondrogenic media had significantly higher storage, loss and complex modulus values than other samples (Figure S1).

Loss modulus results’ (G’) magnitude was significantly lower compared to storage modulus (G”; i.e., PCL/RAD scaffolds in chondrogenic media G’ = 20.28 *±* 1.87 kPa and G” = 3.23 *±* 0.95 kPa), suggesting that scaffolds and encapsulations are much more elastic than viscous as it happens in native cartilage. Additionally, cultured scaffolds’ loss modulus results were not significantly higher compared to the non-treated scaffolds. For the complex modulus, this being the overall resistance of the construct undergoing deformation (combination of elastic plus viscous components), it was observed that an increase of the mechanical resistance was mainly provided by construct’s viscous component.

**Figure 4:**
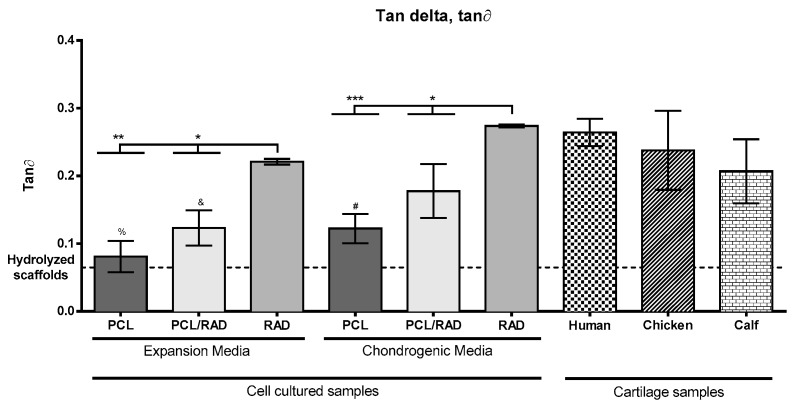
Mechanical properties characterization of PCL, PCL/RAD and RAD constructs under expansion or chondrogenic media culture for 30 days. Tan Delta (tan∂), % stands for statistical differences compared to human (****), chicken (***) and calf (**), and stands for statistical differences with human (**) and chicken (**), # stands for statistical differences with human (**) and chicken (**). Small pieces of human, chicken and calf articular cartilage were measured in the same conditions (Statistical differences are indicated as: *p < 0.05, **p < 0.01 and ***p < 0.001 one-way ANOVA). n = 3.

Tan Delta (tan∂) corresponds to the ratio between loss and storage modulus. A viscoelastic ratio higher than 1 means that the material is more viscous rather than elastic. Native cartilage samples gave the ideal ratio values from which all other samples were compared (human 0.26 *±* 0.02; chicken 0.24 *±* 0.06; calf 0.21 *±* 0.05, Figure 4).

Non-treated samples showed ratios about three times lower than native cartilage and also lower than cultured scaffolds (0.07 *±* 0.01), meaning these are significantly more elastic than viscous. Cell cultured samples of PCL and PCL/RAD conditions from expansion medium and PCL from chondrogenic medium showed significantly lower tan∂ values than native cartilage. No significant differences were found between PCL/RAD and PCL samples between media conditions, although a clear tendency is observed in which PCL/RAD samples have higher viscoelastic ratios than PCL ones. Furthermore, chondrogenic samples also have higher viscoelastic ratios compared to expansion medium ratios. Significant differences were found in viscoelastic ratio between PCL/RAD (0.18 ± 0.04) and RAD (0.27 *±* 0.00) samples in both media conditions, but in chondrogenic medium, neither PCL/RAD nor RAD samples showed significant differences with native cartilage samples. Nevertheless, RAD samples storage and loss modulus values were significantly higher than native cartilage.

### 3.5. Expression of Chondrogenic and Hypertrophic Biomarkers Within the Constructs

Several chondrogenic and hypertrophic biomarkers were analyzed at a genomic and proteomic level to assess chondrogenic differentiation.

**Figure 5:**
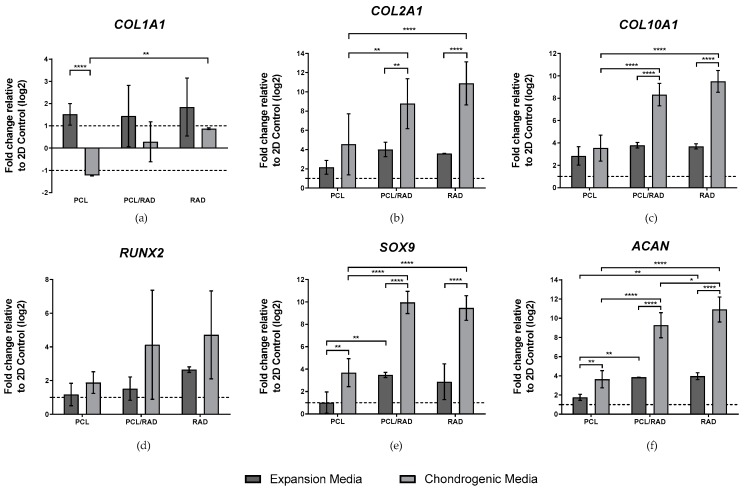
Gene expression of chondrogenic and hypertrophic markers for PCL, PCL/RAD and RAD samples under expansion or chondrogenic media for 30 days. (**a**) Collagen type I (COL1A1), (**b**) collagen type II (COL2A1), (**c**) collagen type X (COL10A1), (**d**) RUNX2, (**e**) SOX9 and (**f**) aggrecan (ACAN) were determined through qRT-PCR. Ct values relative human beta actin (ACTB) were obtained and reported as fold increase (ΔΔCt) relative to 2D cultures (Statistical differences are indicated as: *p < 0.05, **p < 0.01, ***p < 0.001 and ****p < 0.0001, two-way ANOVA). n = 3.

Following 30-day culture, we assessed gene expression of key markers by qRT-PCR. The markers included COL1A1, COL2A1 and COL10A1, RUNX2, SOX9 and ACAN with ACTB as the housekeeping gene (Figure 5). Cells grown as 2D monolayers in expansion medium were used as control samples to which relative fold changes were quantified. Cells grown in PCL, PCL/RAD and RAD scaffolds and cultured in expansion medium had increased expression of COL1A1 (Figure 5a), compared to the 2D control. On the other hand, samples cultured in PCL in chondrogenic medium showed significant down-regulation of COL1A1, correlating with previous reports in articular cartilage regeneration [5,39]. COL2A1 expression (Figure 5b) was significantly up-regulated in all samples both in expansion and chondrogenic media compared to 2D samples. PCL/RAD and RAD chondrogenic scaffolds showed significant differences compared to the scaffolds cultured in expansion medium, suggesting that the chondrogenic medium induced cells to undergo chondrogenic differentiation. Moreover, PCL/RAD and RAD chondrogenic scaffolds expressed higher levels of COL2A1 compared to PCL scaffolds in chondrogenic medium, also suggesting the key role of RAD16-I peptide in hMSCs differentiation. COL10A1 gene expression profile was also upregulated in both media compositions compared to 2D samples (Figure 5c). Again, as observed for COL2A1, PCL/RAD and RAD scaffolds under chondrogenic conditions show higher levels of COL10A1 expression compared to same scaffolds in expansion medium. In addition, PCL/RAD and RAD scaffolds cultured in chondrogenic medium expressed significantly higher amounts of COL10A1 compared to PCL scaffolds. These results are contradictory with the native articular cartilage structure, which does not contain this type of collagen (hypertrophic marker). Lower RUNX2 expression (Figure 5d) was observed compared to COL10A1 and especially to COL2A1. Although there are important expression differences of this gene in PCL/RAD and RAD samples in chondrogenic medium compared to the expansion one, no statistical differences were observed between conditions. Finally, SOX9 and ACAN (Figure 5e,f) display similar gene expression profiles as for COL2A1 and COL10A1, where their expression was up-regulated in most of the conditions. Significant differences were obtained between chondrogenic medium conditions PCL/RAD and RAD compared to PCL scaffolds for all the studied genes. In addition, significant differences were also observed when chondrogenic medium cultured PCL/RAD and RAD scaffolds were compared to the ones cultured in expansion medium, suggesting medium composition and the peptide presence as the two main factors for cell differentiation into chondrocytes.

**Figure 6:**
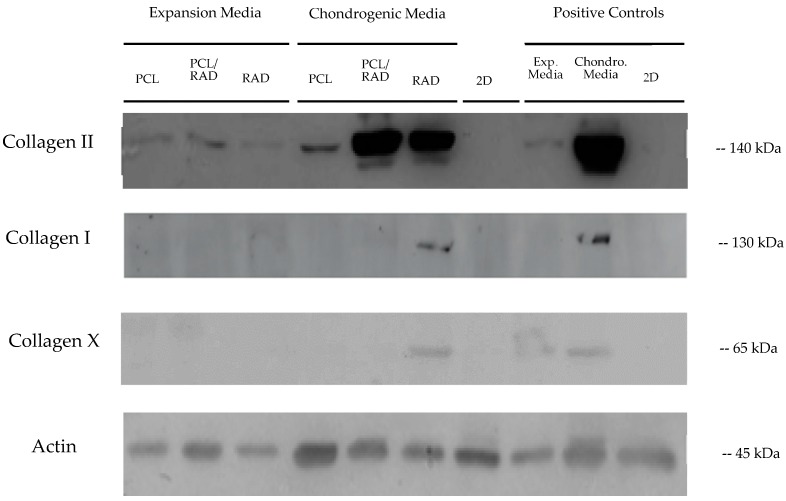
Western Blot results from the human mesenchymal stem cells (hMSCs) assay after 30 days of culture. The expression of three chondrogenic and hypertrophic markers was analyzed in expansion and chondrogenic media, in 2D, and in positive control samples. Actin was used as a control protein. Representative image obtained from two different blots. n = 3.

Protein analysis was performed to correlate gene expression results with protein production. We observed that cultures in PCL scaffolds had low expression of collagen type II (MW 140kDa). The composite scaffolds PCL/RAD and RAD encapsulations had high expression of collagen type II in chondrogenic medium (Figure 6). This suggests RAD16-I is a key contributor to matrix deposition. Furthermore, it was observed from those samples a secondary band below 140 kDa, indicating presence of different immunoreactive collagen type II isoforms. Mature collagen type I bands (MW = 130 kDa) and collagen type X (MW = 65kDa) were only found in RAD constructs and in RAD positive control cultured in chondrogenic medium (Figure 6). Band thickness and intensity were substantially smaller than for collagen type II.

**Figure 7:**
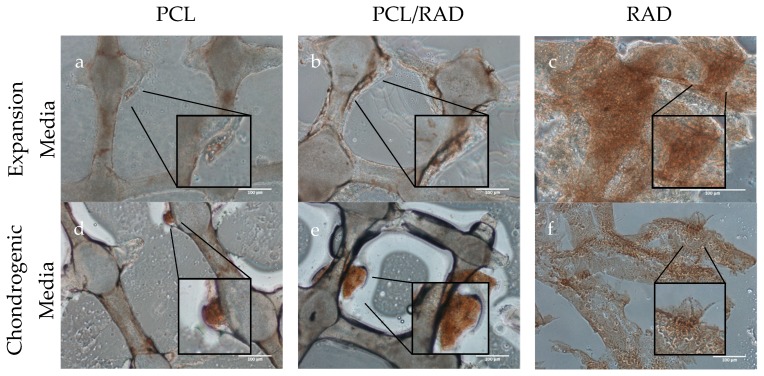
Immunohistochemistry composite picture of collagen type I staining. (**a**–**c**) PCL, PCL/RAD and RAD samples cultured in expansion medium. (**d**–**f**) PCL, PCL/RAD and RAD samples cultured in chondrogenic medium. Scale bar = 100 µm. n = 3.

To further characterize protein expression, samples were immunohistochemically stained for collagen types I and II. PCL samples (Figure 7a,d) showed no differences between culture media composition. Collagen type I staining showed qualitative differences between expansion and chondrogenic media especially for PCL/RAD conditions (Figure 7b,e). Regarding RAD samples, both culture media conditions showed an intense staining suggesting collagen type I deposition (Figure 7c,f,i).

**Figure 8:**
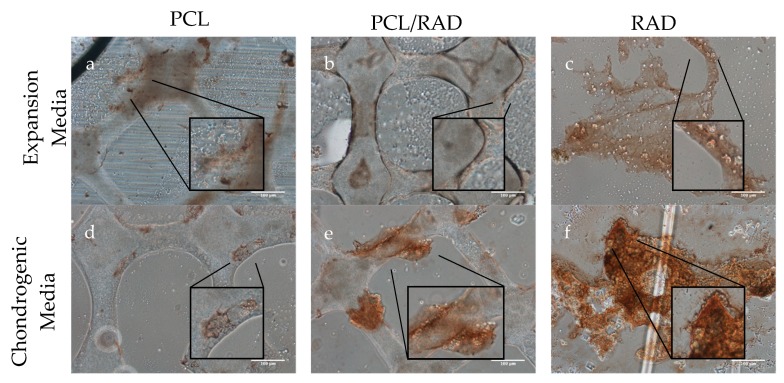
Immunohistochemistry composite picture of collagen type II staining. (**a**–**c**) PCL, PCL/RAD and RAD samples cultured in expansion medium. (**d**–**f**) PCL, PCL/RAD and RAD samples cultured in chondrogenic medium. Scale bar = 100 µm. n = 3.

Immunolocalization of collagen type II showed lower expression of the protein in scaffolds cultured in expansion media compared to the chondrogenic medium ones. No differences in collagen type I intensity were observed in PCL samples for both media conditions (Figure 8a,d). PCL/RAD samples in chondrogenic medium, showed collagen type I staining in cells and ECM aggregates, which was higher compared to expansion medium samples (Figure 8b,e). RAD condition cultured in chondrogenic medium showed higher levels of collagen type I than scaffolds culturing in expansion medium, supporting the ECM deposition hypothesis in chondrogenic conditions (Figure 8c,f). Higher levels of collagen type I were observed in RAD16-I fibers cultured in chondrogenic medium compared to expansion medium.

## 4. Discussion

The increasing incidence of osteo-degenerative and chondro-degenerative diseases due to different factors has arisen as a major problem prompting efforts in developing more efficient strategies to face these pathologies [40,41,42]. To address this problem, we developed a novel 3D bioengineered biocomposite as a regenerative tool to treat chondrogenic defects. Our 3D biocomposite produces cartilage-like tissue through long-term culture and differentiation of mesenchymal stem cells into chondrocytes. The 3D polymer biocomposite is fabricated by 3D printing polycaprolactone scaffolds [43,44], enhanced by the addition of RAD16-I self-assembling peptide [20]. The presented scaffolds mimic the mechanical properties of the native cartilage and provide cells with a microenvironment promoting their viability, proliferation and differentiation. Moreover, scaffold integrity is barely affected by NaOH hydrolysis treatment, as high molar mass of linear PCL chains makes the polymer more hydrophobic, avoiding NaOH to diffuse easily throughout it [45]. This allows only surface hydrolytic cleavage to release carboxyl groups and decrease scaffolds’ hydrophobicity facilitating cell adhesion [46].

Materials cytotoxicity assessment results, following the ISO 10993-5:2009, showed no significant differences in percentage of cell viability at all timepoints, indicating that neither the PCL scaffolds nor the RAD16-I peptide are toxic for cells and that chloroform was successfully removed from PCL scaffolds. Interestingly, only cells cultured in RAD peptide conditioned medium at 72 h exhibited significant higher levels of cell viability (%), confirming the proliferation-enhancing potential of RAD16-I self-assembling peptide [47].

Scaffold morphological characterization showed a cell–cell-interaction-enabling role of RAD16-I self-assembling peptide nanostructure within PCL scaffolds. These peptide networks, allow a better cellular distribution among the construct, creating a denser structure, which can promote cellular signaling, and improved chondrogenic differentiation in presence of pro-chondrogenic factors, as has been previously shown [20,33,37,38]. Micron-sized fibers and a uniformly porous scaffold allows the cells to sense the matrix as a three-dimensional microenvironment [48,49]. Porous scaffold promotes cell–cell and cell–peptide interactions [50] and cell migration [51], while allowing a correct perfusion of nutrients and oxygen [52,53]. MSCs cultured in chondrogenic medium rather than expansion medium in PCL/RAD biocomposite had lower proliferation rate but deposited denser ECM, confirming the chondrogenic potential of the differentiation media [54].

It is known that cell seeding density has an influence in proliferation, migration, differentiation and gene expression [55]. Too high or low cellular densities can negatively affect these processes depending on cell type [56,57,58]. Nevertheless, the environment that cells feel and interact with (medium composition, two or three-dimensional culture and scaffold type), has also an important role in these processes [55]. In that sense, even though lower cell densities were used in RAD encapsulations, culture medium composition and RAD16-I scaffold properties, greatly affected their behavior (differentiation and biomarkers expression) and cross-talk.

RAD constructs showed viscoelastic modulus values which perfectly matched human articular cartilage results. However, other mechanical properties, including storage and loss modulus, showed significant differences with native cartilage and even higher differences compared to PCL and PCL/RAD scaffolds. This means that the peptide constructs are more elastic and viscous than native cartilage, explained by their soft and highly elastic fibers in wet conditions, which could entail non-desired mechanical responses as joint replacements [59]. Moreover, after mechanical testing, RAD composites were unable to recover the initial shape, excluding the possibility to repeat measurements with the same sample and therefore, impeding the enforcement of constant and repeated stresses, leading to low durability and less resistance to repetition of compressive loads and deformations when placed in patients’ joints, whereas 3D printed scaffolds were able to return to their initial shape to reload the stress.

On the other hand, by including RAD in the PCL scaffolds, a strong correlation was found between mechanical testing, gene expression and protein deposition results, suggesting PCL/RAD to be the most effective condition for articular cartilage tissue regeneration. With the combination of these two materials cultured under chondrogenic conditions, we increased the viscoelastic modulus towards the level of human articular cartilage, without affecting storage and loss modulus and improving the mechanical properties compared to previous work [20,37]. Moreover, in this condition it was observed a strong expression of the key chondrogenic genes such as COL2A1, SOX9 or ACAN and the deposition of collagen type II, whilst collagen type I deposition was not observed. These results are consistent with recent publications on cartilage regeneration using bone marrow hMSCs with self-assembling peptides such as RAD16-I or KLD12 [37,60] and either mono- or copolymeric hydrogels of polyethylene glycol (PEG) [61,62], chitosan [63,64], agarose [65,66] and alginate [67,68,69]. In addition, although a strong COL10A1 gene expression in PCL/RAD biocomposites was observed, no translation of this gene to the collagen type X protein was observed, avoiding hypertrophic processes described in previous work [70,71,72]. These facts support the hypothesis of articular chondrogenic differentiation where collagen types I and X expression ceases, and collagen type II expression is over-regulated, whereas the opposite occurs in fibrous cartilage [73]. RAD encapsulations cultured in chondrogenic media showed similar gene and protein expression patterns compared to PCL/RAD constructs, highlighting the important role of the peptide in cell differentiation due to the nanostructure provided to cells [60]. However, PCL scaffolds demonstrated poor viscous properties, but, when both polymers were combined in PCL/RAD constructs, viscoelastic properties increased to native cartilage values, showing an important role of RAD16-I self-assembling peptide in providing a viscous component to constructs. Additionally, the absence of RAD16-I self-assembling peptide in constructs, had a strong impact on the gene expression profile in PCL samples compared to PCL/RAD. In both expansion and chondrogenic culture media conditions gene downregulation was observed, with significantly less expression than conditions with RAD16-I peptide, and small deposition of chondrogenic proteins occurred. This on the other hand also affected the mechanical properties, demonstrating again RAD16-I’s key role in providing cells a nanostructure similar to the native microenvironment, which in turn will trigger chondrogenic differentiation, gene and protein expression and improve mechanical properties. Significant differences were found comparing media compositions [54], PCL/RAD constructs in chondrogenic medium had higher viscoelastic properties than those in expansion medium, possibly due to the matrix remodeling carried out by differentiated cells. This suggests a strong correlation between the chondrogenic medium composition inducing cell differentiation and the expression of articular cartilage genes and ECM deposition, which would lead to the enhancement of the mechanical properties of these PCL/RAD scaffolds [60]. Gene expression of COL2A1, SOX9 or ACAN genes and protein deposition were higher under chondrogenic conditions, confirming the differentiation potential of chondrogenic media. The expression of chondrogenic markers followed by articular cartilage ECM deposition might also have improved the viscoelastic properties of the constructs, making their properties closer to native cartilage.

## 5. Conclusions

Articular cartilage tissue regeneration has been demonstrated to be a complex and long-lasting process. Herein, we designed and produced a bioengineered platform combining 3D-printed PCL scaffolds and RAD16-I self-assembling peptide, obtaining a long-lasting biocomposite for complete joint tissue regeneration. Our results demonstrated that this biocomposite effectively drives mesenchymal stem cell differentiation into chondrocytes by providing cells with a suitable microenvironment with peptide nanofibers and chondrogenic-factor supplemented medium. The differentiated chondrocytes commence articular cartilage ECM production. PCL scaffolds in combination with RAD16-I and the ECM produced by cells increased mechanical properties to values similar to those of native human articular cartilage values. These results suggest our biocomposite could be a potential clinical tool for articular tissue regeneration, as a more efficient way to address chondrodegenerative pathologies.

## Figures and Tables

**Table 1:** qRT-PCR primer sequences.

Gene		Sequence (5’-3’)	Size (bp)
COL1A1	Forward	5′-AGACGGGAGTTTCTCCTCGG-3′	20
	Reverse	5′-CGGAGGTCCACAAAGCTGAA-3′	20
COL2A1	Forward	5′-ATGACAATCTGGCTCCCAAC-3′	20
	Reverse	5′-CTTCAGGGCAGTGTACGTGA-3′	20
COL10A1	Forward	5′-CCAATGCCGAGTCAAATGGC-3′	20
	Reverse	5′-GGGGGAAGGTTTGTTGGTCT-3′	20
SOX9	Forward	5′-CAGACGCACATCTCCCCCAA-3′	20
	Reverse	5′-GCTTCAGGTCAGCCTTGCC-3′	19
ACAN	Forward	5′-TGGTGATGATCTGGCACGAG-3′	20
	Reverse	5′-CGTTTGTAGGTGGTGGCTGT-3′	20
RUNX2	Forward	5′-GGTTCAACGATCTGAGATTTGTGGG-3′	25
	Reverse	5′-CACTGAGGCGGTCAGAGAACAAACTAG-3′	27
ACTB		PPH00073G-200, Qiagen	-

## Supplementary Materials

The supplementary materials are available online at https://www.mdpi.com/2218-273X/10/1/52/s1.
