# Supplementary figures and images for: Development of a Three-Dimensional Bioengineered Platform for Articular Cartilage Regeneration
Source: Biomolecules. 2019 Dec 28;10(1):52. doi: 10.3390/biom10010052 (PMC7023234; doi:10.3390/biom10010052)

A

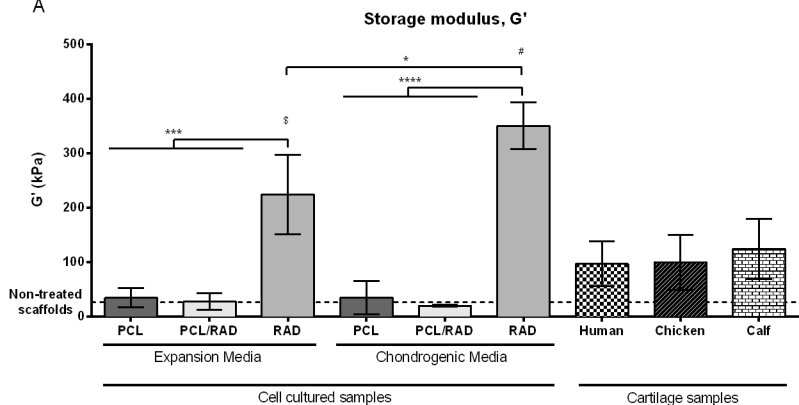

B

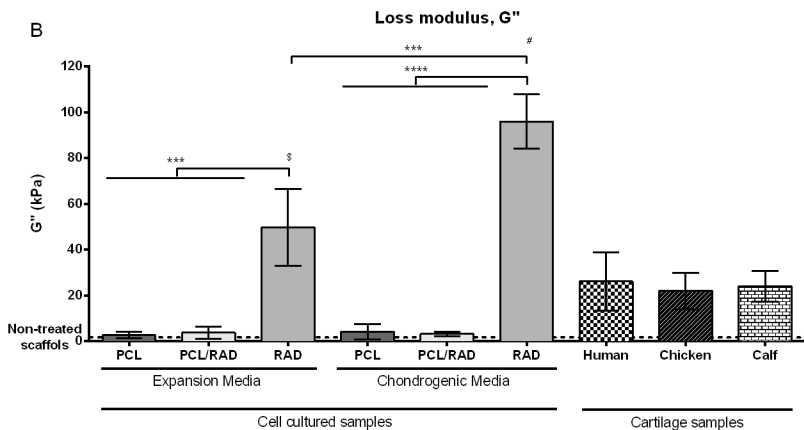

C

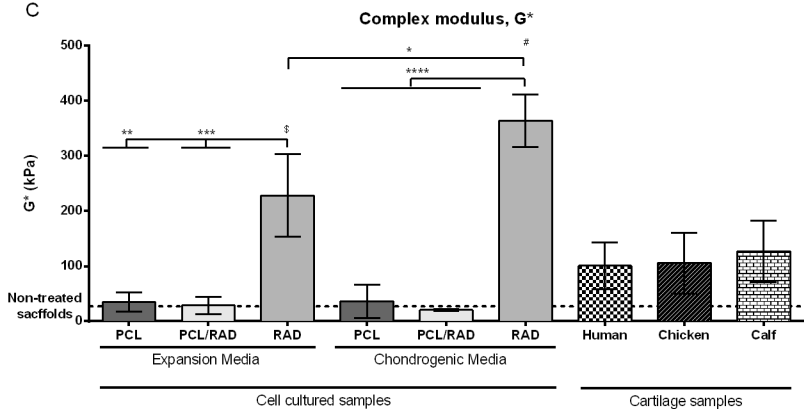

Supplement: Supplementary file 1 [file biomolecules-10-00052-s001.pdf]
